# Correlation of sleep quality and cardiac autonomic modulation in hemodialysis patients

**DOI:** 10.5935/1984-0063.20200126

**Published:** 2022

**Authors:** Erika Ribeiro Carneiro, Luana Anaisse Azoubel, Raimunda Carneiro Dias, Carlos José Dias, Emanuelle Sousa Sá, Dyego Araujo Brito, Natalino Salgado Filho, Elton Freitas Santos, José Hermógenes Rocco, Cristiano Teixeira Mostarda, Mario Bernardo Filho

**Affiliations:** 1 Universidade Federal do Maranhão, Hemodialysis - São Luis - Maranhão -Brazil.; 2 Universidade Federal do Maranhão, Center for Prevention of Kidney Diseases of the University Hospital -Sao Luis - Maranhão - Brazil.; 3 Universidade Federal do Maranhão, Physical Education Department - Sao Luís - Maranhão - Brazil.; 4 Universidade do Estado do Rio de Janeiro UERJ, Laboratory of Mechanical Vibrations and Integrative Practices, Department of Biophysics and Biometrics, - Rio de Janeiro -Rio de Janeiro - Brazil.; 5 Laboratório da adaptações cardiorrenais ao exercício físico (LACE), UFMA -Campus Pinheiro; 6 Universidade Estadual do Rio de Janeiro -UERJ - Rio de Janeiro - RJ - Brazil.

**Keywords:** Sleep Quality, Hemodialysis, Autonomic Nervous System Diseases

## Abstract

**Objectives:**

Sleep disorders in patients on hemodialysis are frequent, but few studies correlate these disorders with autonomic dysfunction in these patients. This study aimed to verify whether clinical and laboratory variables and heart rate variability are associated with worse sleep quality verified by the Pittsburg subjective scale in patients on hemodialysis.

**Material and Methods:**

A cross-sectional study was performed on forty-eight patients. Epidemiological, clinical, and laboratory data were collected. After were performed by recording the heart rate variability and applied Pittsburg questionnaires, Beck anxiety index (BAI), and Beck depression index (BDI). The global PSQI score >5 indicates that a person is a poor sleeper, the patients were divided according to the scores in the Pittsburg questionnaire into good and poor sleepers and the differences between all variables were analyzed.

**Results:**

Forty-eight patients were evaluated and the prevalence of 68.7% (n=33) of poor sleep quality was verified. From the depression and anxiety questionnaires, it was found that only 18.7% (n=9) had criteria for depression. In the analysis of the sympathetic dysfunction parameters, it was found that in the group with good sleep quality in the frequency domain (HFm^2^) and the LFnu in the group with worse sleep quality. There was a positive correlation between sleep quality scores the anxiety and depression scores. It is also verified that the variables LFnu had a positive correlation with higher scores of quality of sleep and HFnu had a negative correlation with the highest scores of quality of sleep.

**Conclusion:**

In patients undergoing hemodialysis, the poorest quality of sleep is correlated with worse cardiac autonomic modulation as well as higher scores on the depression and anxiety scales.

## INTRODUCTION

Currently more than 2.5 million people are on renal replacement therapy worldwide and it is estimated that by 2030 this number is expected to double^1,2^. Among the many problems in physiology faced by patients on hemodialysis, sleep disorders (SD) are common conditions and frequently underdiagnosed in clinical practice, therefore it is important to investigate through interviews and questionnaires such as the one in Pittsburgh and Epworth to direct better use of the gold standard polysomnography, which is still not very accessible in our country. They are usually more frequent in hemodialysis than in chronic kidney disease (CKD) non-dialysis dependent, and it has been verified in studies that there may be a worsening after starting dialysis therapy^3,4^.

The prevalence mean of SD is 44% (20-83%)^4,5^ and may be subdivided into insomnia, sleep apnea, restless legs syndrome and excessive daytime sleepiness or be related to depression (another condition prevalent in these patients)^6^.

Some sleep disorders are linked to worsening hypertension and cardiovascular diseases such as obstructive sleep apnea and restless legs syndrome^7^; however, in other disorders this association is not clear. An important multicenter study with 11,351 patients from 7 countries found a 49% prevalence of sleep disorders in patients on hemodialysis, associated with lower quality of life scores, physical inactivity, use of some medications, cardiovascular disease, and increased serum phosphorus levels^8^.

Concerning the autonomic nervous system in chronic renal patients, different studies demonstrate an imbalance between sympathetic and parasympathetic activity with alteration of tone and neural refexes, leading to the deleterious effects of sympathetic hyperactivity, which may contribute to the increased incidence of sudden death in this population^9,10,11^.

The biological effects of CKD on sleep can be partially explained by the hyperreactivity of the sympathetic nervous system, decreased barorefex activity leading to continuous stimulation of the renin-angiotensin-aldosterone system and non-dipper pattern of blood pressure commonly seen in chronic renal patients, being associated worse cardiovascular outcomes^12,13,14,15,16^.

Sympathetic hyperreactivity may partially explain episodes of intradialytic hypertension and refractory hypertension. Decreased heart rate and barorefex function variability has been associated with increased morbidity and mortality in hemodialysis patients^17^. It is postulated that one of the mechanisms of sleep disorders in chronic renal patients may also be due to increased activation of the sympathetic nervous system^18^.

Many studies have been done evaluating sleep disorders in hemodialysis patients, but few studies that correlate these disorders with autonomic dysfunction in these patients. The aim of this study was to verify whether clinical and laboratory variables and heart rate variability are associated with worse sleep quality verified by the Pittsburg subjective scale in patients on hemodialysis.

## MATERIAL AND METHODS

### Sample

A cross-sectional study was performed in 152 patients undergoing hemodialysis therapy in two centers of hemodialysis at Maranhão, from October 2018 to June 2019. From this total 66 were included in previous research, of which 48 completed all evaluations and questionnaires. The study was authorized by the ethics committee (CEP), CAAE: 99019318.0.0000.5086.

The survey included only adults’ patients who had been enrolled in the service for at least 3 months. Patients with heart disease (arrhythmias, coronary disease, and heart failure) debilitating active or chronic lung disease and patients with neurological deficits were not included. Epidemiological, clinical and laboratory data were collected from individuals with chronic kidney disease on hemodialysis and electrocardiogram were performed to analyze heart rate variability.

After signing the consent form, patients answered a questionnaire with demographic information, clinical history (gender, age, residence, education, and income) and information about hemodialysis sessions (frequency and duration of sessions per week, time in hemodialysis, and shift). Physical examination was done verifying weight, height, body mass index (BMI), and blood pressure. Blood samples were collected for the following exams: hemoglobin, calcium, phosphate, urea, glycemia, HDL cholesterol, LDL cholesterol, parathormone, alkaline phosphatase, ferritin, potassium, albumin, and creatinine and analyzed in institution’s laboratory. The Kt/V - number used in nephrology to calculate the dialysis dose - should be above 1.2, where: K (urea kinetics), T (time), and V (volume of urea distribution). In another day was performed by recording the heart rate variability (HRV) and applied Pittsburg questionnaires, Beck anxiety index (BAI) and Beck depression index (BDI).

### Heart rate variability (HRV)

To extract signals to HRV was realized an eletrocardiogram 12-leads utilizing the device micromed wincardio (Micromed – WinCardio, v. 6.1.1 software) to obtain R-R intervals. The exam was realized in a silent room, the patient was instructed not to speak during exam and remain the supine position during 10 minutes while the exam was performed. After exam the intervals R-R series were extrated in format txt using software wincardio, HRV indices were analyzed using Kubios HRV software, version 3.2 (Biosignal Analysis and Medical Imaging Group, Kuopio, Finland).

The software converts signals R-R to digital electrocardiogram in variables to examine autonomic nervous system function. All dates were disposable after correct about filter artefacts and were used SDNN (standard deviation of RR intervals) and RMSSD (Root mean square of successive RR intervals differences) in the time-domain. In frequency-domain were utilized low frequency (LFms^2^) (LFnu-nor malized) and high frequency (HFms^2^) (HFnu-normalized) bands in milliseconds and percentage, that represents predominantly sympathetic and parasympathetic modulation, respectively, and LF/HF ratio as representative vagal sympathetic balance^19,20^.

### Pittsburg sleep quality index

Sleep quality was analyzed based on the instrument called Pittsburgh sleep quality index (PSQI), elaborated by Buysse et al. (1989)^23^, tested and validated in Brazil^24^. The questionnaire, applied in this study in the form of an interview, consists of 19 questions regarding quality and sleep disorders in the last month. The questionnaire evaluates seven components of sleep: subjective quality, sleep latency, sleep duration, sleep efficiency, sleep disorders, medication use, and daily dysfunction. For each component, the score can vary from 0 to 3, with the upper limit representing greater component impairment. The total sum of the scores generates results ranging from 0 to 21. The global PSQI score >5 indicates that a person is a ‘poor sleeper’ having severe difficulties in at least two areas or moderate difficulties in more than three areas.

### Beck anxiety index

The BAI is a self-reported scale that measures the intensity of anxiety symptoms. The scale was built based on several auto-reporting instruments used in the CCT (Center for Cognitive Therapy), from which items were selected to compose the Inventory. The inventory consists of 21 items with descriptive statements of anxiety symptoms and should be evaluated by the subject with reference to himself, on a scale of four points, which, according to the manual reflect levels of increasing severity of each symptom: 1) absolutely not; 2) slightly: did not bother me much; 3) moderately: was very unpleasant, but I could bear it; 4) severely: could hardly bear it^25^.

### Beck depression index

The BDI is composed of 21 items that include the cognitive, affective, behavioral, and somatic components of depression. Each item contains four statements that vary in intensity (0 to 3), with the individual indicating which of the four statements best describes his symptoms. The final score is obtained by adding together the 21 items that make up the scale, resulting in the following normatization, according to the Cognitive Therapy Center: (a) no depression or minimum depression: final scores less than 11 points; (b) mild to moderate depression: final scores between 12 and 19 points; (c) moderate to severe depression: final scores between 20 and 35 points; and (d) severe depression: final scores between 36 and 63 points^25^.

### Statistical analysis

All data were presented in mean and standard deviation. The Shapiro-Wilk test was applied to verify the normality of the data. The parameters of interest were analyzed using unpaired Student’s t-test or Mann-Whitney U test depending on normality. The chi-square test or Fisher’s test was applied to verify if there is an association between the qualitative variables. Pearson’s and Spearman’s correlations were verified on the variables. The results were considered statistically significant for the value *p*<0.05. For data analysis, GraphPad Prism version 8.0.0 for Mac OSx, GraphPad Software, San Diego, California, U.S.A., www.graphpad.com was used.

## RESULTS

Forty-eight patients were evaluated and the prevalence of 68.7% (n=33) of poor sleep quality was verified. From the depression and anxiety questionnaires, it was found that only 18.7% (n=9) had criteria for depression (mild to severe) and 22.9% (n=11) mild to severe anxiety. The most frequent etiology found was chronic glomerulonephritis 29.1% (n=14) followed by undetermined cause 25% (n=12) reflecting the relatively young population (mean age 48.32±12.37 years) of this study. Hypertensive nephrosclerosis and nephropathy diabetic were found in 20.8% (n=10) and 10.4% (n=5) of patients, respectively. The mean time in hemodialysis was 61.44^±^45.8 months. The mean BMI was 23.8±4.0. There were no significant differences regarding the use of antihypertensives, with 4 patients (26.6%) in the good sleepers group and 11 patients (33.3%) in the poor sleepers group using ARB or ACE inhibitors (*p*=0.23) and 5 patients (33.3%) in the good sleepers group and 10 patients (30.3%) in the poor sleepers group using betta-blockers (*p*=0.99).

The socio-demographic variables analyzed were 70% (n=33) of the patients had more than 8 years of formal education, 79% (n=38) of the patients were from the city where they were on dialysis, and 75% (n=36) of the patients were on the night shift, and average monthly income of 397 dollars, when we divided the patients as to the quality of sleep these variables did not show interference (Table 1).

**Table 1.** Clinical and demographic characteristics of good sleepers compared with poor sleepers in hemodialysis.

Variable	Good sleepers PSQI < 5 (15)	Poor sleepers PSQI >5 (33)	p-value
**BAI**	5.66±6.68	9.8±9.09	0.12
**BDI**	5.00±3.63	11.65 ±8.68	0.004*
**Sleep quality**	3.60±1.35	8.43±2.67	-
**Vintage HD**	45.4±28.6	65.7±51.6	0.32
**Age (years)**	46.4±14.3	49.2±11.9	0.48
**Presence of diabetes**	2/15 13.3%	5/33 15.1%	ns
**Women/men**	8/ 7	14/ 18	0.75
**BMI**	23.7±3.83	24.3±4.15	0.65
**SBP (mmHg)**	132.3±18.8	138.3±19.5	0.32
**DBP (mmHg)**	84±10.6	86.3±10.8	0.50
**6-min WT (meters)**	412.1±108.9	444.3±96.4	0.28
**Scholarity**			
**<8 years**	2/15 13.3%	12/33 36.3%	0.17
**>8 years**	13/15 86.6%	21/33 63.6%	
**Distance of centre dialysis Near (same city)**	13/15 86.6%	25/33 75.7%	0.47
**Far (different city)**	2/15 13.3%	8 /33 24.2%	0.07
**Shift Evening**	1/15 6.6%	11/33 33.3%	
**night**	14/15 93.3%	22/33 66.6%	

Notes: PSQI = Pittsburgh sleep quality index; BAI = Beck anxiety index; BDI = Beck depression index; BMI = Body mass index; SBP = Systolic blood pressure; DBP = Diastolic blood pressure; 6-min WT (6 minutes walking test); *Mann Whitney; #Fisher test.

Clinical variables such as time on hemodialysis, age, gender, BMI, blood pressure, presence of diabetes mellitus, and the cardiopulmonary capacity inferred by the walking test did not differ between groups with good and bad sleep quality (Table 1). Among the laboratory tests, no difference was observed either (Table 2). In the analysis of the sympathetic dysfunction parameters, it was found that in the group with good sleep quality in the frequency domain (HFm2) it shows higher integrity of the parasympathetic nervous system and in the LFnu it shows higher sympathetic modulation in the group with worse sleep quality (Table 3). In time domain the RMSSD index was numerically lower in the group with higher PSQI, but not statistically significant.

**Table 2.** Laboratory data of good sleepers compared with poor sleepers in hemodialysis.

Variable	Good sleepers PSQI < 5 (15)	Poor sleepers PSQI >5 (33)	p-value
**Phosphate (mg/dL)**	4.59±1.05	4.58±1.01	0.97
**Calcium (mg/dL)**	9.02±0.74	8.90±0.79	0.63
**Calcium x phosphate product**	41.8±11.9	40.78±9.59	0.74
**Urea (mg/dL)**	121.5±37.6	131.1±34.4	0.39
**Potassium mmol/L**	4.89±0.79	5.16±0.93	0.33
**Hemoglobin (g/dL)**	11.5±1.60	11.15±1.82	0.32
**Glycemia (mg/dL)**	91.7±20.9	107.7±37.9	0.31
**HDL cholesterol (mg/dL)**	39.2±13.9	40.3±15.2	0.81
**LDL cholesterol (mg/dL)**	91.7±19.5	99.5±29.1	0.44
**PTH pg/ml**	675.6±443.9	472.9±413.5	0.08
**Alkaline phosphatase U/l**	161.5±55.8	152.9±137	0.07
**Ferritin (ng/mL)**	395.9±179.7	618.9±468.8	0.18
**albumin (g/dL)**	4.26±0.41	4.18±0.57	0.38
**KTVe**	1.40±0.26	1.27±0.38	0.23

Notes: PTH = Parathyroid hormone; PSQI = Pittsburgh sleep quality index; KTVe K (urea kinetics), T(time), and V (volume of urea distribution).

**Table 3.** Variables of heart rate variability (HRV) of good sleepers compared with poor sleepers in hemodialysis.

Variable	Good sleepers PSQI < 5 (15)	Poor sleepers PSQI >5 (33)	p-value
**mean RR (ms)**	798.9±91.4	781.5±114.2	0.60
**SDNN (ms)**	19.8±7.56	16.13±6.22	0.15
**RMSSD (ms)**	15.3±6.26	11.63±6.63	0.07
**LF (ms²)**	66.5±53.2	62.3±64.5	0.48
**HF (ms²)**	109.6±101.5	58±63.7	0.02^*^
**LF/HF**	1.21±1.05	2.55±2.64	0.05
**LFnu**	45.9±19.8	59.3±21.1	0.04^#^
**HFnu**	53.9±19.7	43.1±22.1	0.11

Notes: PSQI = Pittsburgh sleep quality index; Mean RR = Mean intervals R-R; SDNN = Standard deviation of RR intervals; RMSSD = Root mean square of successive RR intervals differences; LF ms2 = Low frequency expressed ms2; LFnu = Low frequency normalized HFms2 - high frequency expressed ms2; HFnu = High frequency normalized; LF/HF= ratio-vagal sympathetic balance; *Mann Whitney test; #Unpaired t-test.

**Figure 1.**
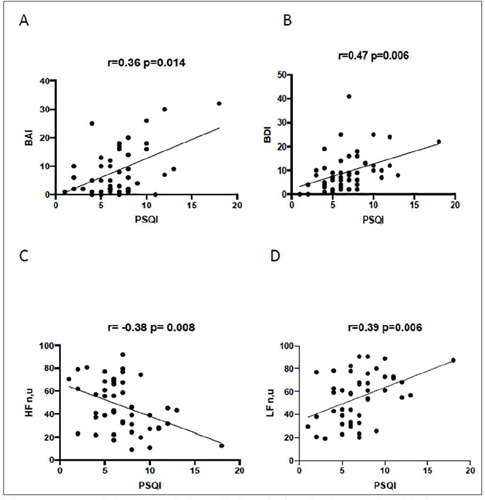
Correlations correlation of the Pittsburgh sleep scale with heart rate variability (HRV) parameters and Beck’s depression and anxiety scales. A) BAI Beck and PSQI B) BDI Beck and PSQI C) HFnu and PSQI D) LFnu and PSQI.

There was a positive correlation of the sleep quality scores with the anxiety and depression scores (Table 4^,^Figures 1A and 1B), demonstrating that these are conditions that are usually part of the picture associated with chronic diseases and that alter the quality of life. It is also verified that the variables of variability of heart rate in frequency domain LFnu (which indicates higher sympathetic modulation) had positive correlation with higher scores of quality of sleep and HFnu (which indicates parasympathetic integrity) had negative correlation as the highest scores of quality of sleep (Table 4^,^Figures 1C and 1D).

**Table 4.** Correlation of the Pittsburgh sleep scale with heart rate variability (HRV) parameters and Beck’s depression and anxiety scales.

Correlation Pittsburg	r	p
**LF nu**	0.39	0.006*
**HF nu**	-0.38	0.008*
**BDI**	0.47	0.006#
**BAI**	0.36	0.014#

## DISCUSSION

Hemodialysis patients have significant impairments in quality of life due to uremic symptoms, time spent on dialysis treatment (usually three times a week and four hours a day), financial difficulties generated by unemployment and early retirement, in addition to the impact due to bone and muscle pain caused by osteoarthritis and physical deformities due to the use of fistulas and catheters. All of these factors favor conditions of anxiety and depression that can impact the sleep quality.

These disorders (anxiety, depression, and sleep disorders), despite extensive literature on the subject, are often underdiagnosed in clinical practice^26^, and sometimes directed anamnesis and questionnaires are necessary, thus studies that correlate the importance of the subject in the impaired quality of life and cardiovascular risk of these patients. The present study did not verify that socio-demographic variables infuenced on the worse quality of sleep. A Turkish study found an association with the patient’s current unemployment^27^, but other studies including a Brazilian study did not find this association^28,29^. Studies show that the poorest quality of sleep in hemodialysis patients was associated with low income^30,31,32,33^. However, this association was not verified in this study, in the state of Maranhão, most patients undergoing hemodialysis have low income and some unemployment insurance or sickness insurance, perhaps explaining the uniformity of the findings^34^.

In previous studies, it appears that sleep quality was related to anemia^35,36^, hyperphosphatemia^37,38^, nutritional status^39^, uremic pruritus, hyperparathyroidism^40^, time on hemodialysis^35^, female^35,41^, age^27,28,41,42^; however, in this study these associations were not verified. A study by Bilgic et al. (2007)^39^ found that those patients with higher malnutrition and inflammation scores had worse sleep quality, in the present study the BMI, albumin, and serum ferritin did not differ between groups.

Although the sample of patients is small, it is observed that in the present study, laboratory and clinical variables were not associated with worse sleep quality, which may be due to the uniformity and availability of treatment of these patients for anemia, bone mineral disease, adequacy of dialysis with better technology in hemodialysis machines, and drug treatment (such as erythropoietin, phosphorus binders, and calcimmetics) involved.

It is observed in more recent studies that among the clinical and laboratory variables, only female gender^41^ and age^41,42^ were related to worse sleep quality. A recent study by Turkish researchers^43^ found an important correlation between sleep quality and the dialysis quality index (good dialysis index).

The association between poor sleep quality and sleep disorders and autonomic dysfunction is already well established in the literature^44,45,46,47^; however, in the hemodialysis population this association is still poorly studied. The normal phases of sleep are closely related to the autonomic nervous system, whereas in the non-REM phase the predominance of parasympathetic establishes a fall in heart rate and blood pressure, favoring cardiovascular protection, although in chronic kidney patients (especially those in chronic kidney phases advanced diseases)^10,11,12^, there is reduced parasympathetic modulation and sympathetic exacerbation, with repercussions on the cardiovascular system (absence of physiological nocturnal fall in blood pressure, left ventricular hypertrophy, and accelerated atherosclerosis)^48^, therefore, it is not surprising to find that in addition to previous autonomic dysfunction, these patients may have their condition aggravated by sleep disorders, anxiety and depression.

This study demonstrated that patients with worse sleep quality had parameters of heart rate variability with greater sympathetic hyperactivity, this being one of the few studies that correlates parameters of autonomic dysfunction in these patients with worse sleep quality^49,50,51^. A study by Chan et al. (2004)^49^ found an improvement in autonomic dysfunction and apnea/hypopnea parameters in polysomnography of patients after switching from conventional to nighttime hemodialysis. Study with 14 Japanese patients found autonomic dysfunction (pNN50 and minor RMSSD) in patients on hemodialysis (regardless of whether hemodialysis was performed on the day of the exam) compared to controls^50^, demonstrating that parasympathetic activity in hemodialysis patients was lower during sleep than in the control group.

In study 2010, using polysomnography and recording of heart rate variability during sleep and wakefulness found that in patients with advanced stage CKD (CKD 4-5) and on hemodialysis, the parasympathetic tone does not increase as it should in the wake transition period for sleep, being one of the first studies to identify autonomic dysfunction during sleep in chronic kidney patients; however, it did not assess whether it would have an impact on sleep quality^52^. Therefore, further studies are needed to verify whether autonomic dysfunction is a cause or consequence of the worse quality of sleep seen in these patients. This study has some limitations because it is a cross-sectional study with a reduced number of patients due to the difficulty in applying the sleep questionnaire and performing the pre-dialysis before hemodialysis session heart rate variability electro. We did not have ambulatory blood pressure monitoring for all patients, mainly due to the protocol difficulty of taking the exam in 44 hours (on the day of dialysis and on the day without hemodialysis). The gold standard for checking polysomnography sleep disorder was not possible for equipment unavailability.

## CONCLUSION

Thus, it can be concluded with this study that the quality of sleep correlates with Beck’s scale of depression and anxiety in hemodialysis patients, therefore being important scales for screening these disorders that impact the quality of life of these patients, thus enables health professionals to act earlier. The worse quality of sleep verified by the Pittsburg sleep scale was also correlated with autonomic dysfunction in these patients, being an important marker of cardiovascular risk.
